# Engulfing Astrocytes Protect Neurons from Contact-Induced Apoptosis following Injury

**DOI:** 10.1371/journal.pone.0033090

**Published:** 2012-03-26

**Authors:** Camilla Lööv, Lars Hillered, Ted Ebendal, Anna Erlandsson

**Affiliations:** Department of Neuroscience, Uppsala University, Uppsala, Sweden; Brigham and Women's Hospital, - Harvard Medical School, United States of America

## Abstract

Clearing of dead cells is a fundamental process to limit tissue damage following brain injury. Engulfment has classically been believed to be performed by professional phagocytes, but recent data show that non-professional phagocytes are highly involved in the removal of cell corpses in various situations. The role of astrocytes in cell clearance following trauma has however not been studied in detail. We have found that astrocytes actively collect and engulf whole dead cells in an *in vitro* model of brain injury and thereby protect healthy neurons from bystander cell death. Time-lapse experiments showed that migrating neurons that come in contact with free-floating cell corpses induced apoptosis, while neurons that migrate through groups of dead cells, garnered by astrocytes, remain unaffected. Furthermore, apoptotic cells are present within astrocytes in the mouse brain following traumatic brain injury (TBI), indicating a possible role for astrocytes in engulfment of apoptotic cells *in vivo*. qRT-PCR analysis showed that members of both *ced* pathways and *Megf8* are expressed in the cell culture, indicating their possible involvement in astrocytic engulfment. Moreover, addition of dead cells had a positive effect on the protein expression of MEGF10, an ortholog to CED1, known to initiate phagocytosis by binding to phosphatidylserine. Although cultured astrocytes have an immense capacity for engulfment, seemingly without adverse effects, the ingested material is stored rather than degraded. This finding might explain the multinuclear astrocytes that are found at the lesion site in patients with various brain disorders.

## Introduction

Astrocytes respond to all forms of CNS insults through a process referred to as reactive astrogliosis. It is well documented that the reactive astrocytes undergo hypertrophy, upregulate intermediate filaments composed of nestin, vimentin and glial fibrillary protein (GFAP) and give rise to the glial scar [1]. However, the importance of astrocytes in the clearing of dead cells following injury remains unclear.

Engulfment of apoptotic cells has traditionally been attributed to professional phagocytes, such as macrophages, microglia and dendritic cells, but recent data suggests that neighboring cells often are responsible for the removal of apoptotic cell corpses. Phagocytosis by tissue-resident, neighboring cells has for example been demonstrated in adult mammary glands and testis [2], [3], [4]. Moreover, non-professional phagocytes are known to be highly involved in clearing the massive number of cells that undergo apoptosis during animal development [5]. It was recently demonstrated that satellite glial cell precursors are the primary phagocytic cells for apoptotic cell corpse removal in the developing mouse dorsal root ganglia [6] and astrocytic glia have been shown to be the main phagocytes in the late nervous system development in *Drosophila* [7].

The occurrence of astrocytes with multiple distinct nuclei has been described in several disorders including stroke, brain tumors, multiple sclerosis (MS) and sporadic Creutzfelt-Jacob disease [8], [9], [10], [11], [12], [13], but the underlying mechanisms for the occurrence of multinucleated astrocytes are unclear. There are a few *in vitro* studies indicating that astrocytes are capable of engulfing cell corpses, but no detailed investigations have been performed [14], [15]. It was however shown by several electron microscopy studies already in the 1970s, that astrocytes can engulf smaller fragments, such as axonal or myelin debris [16], [17], [18], [19], [20], [21], [22]. Furthermore, astrocytes have been shown to clear amyloid deposits *in vitro* [23].

In the present study we demonstrate, by using electron transmission, confocal and time-lapse microscopy, that astrocytes effectively engulf whole dead cells and constantly cleaning up cell corpses following neural scratch injury *in vitro*. By ingesting free-floating cell corpses, the astrocytes protect neurons following injury from contact-induced cell death. Furthermore, we have identified astrocytes with incorporated TUNEL positive cells in an animal model of TBI, indicating a possible role of engulfing astrocytes following acute brain trauma *in vivo*. Even though cultured astrocytes have the ability to incorporate multiple nuclei, the novel staining technique pHrodo™ succinimidyl ester (pHrodo) show that, in contrast to macrophages, the astrocyte ingested material appears to be stored rather than degraded.

## Results

### Neurons, astrocytes and oligodendrocytes respond differently to injury *in vitro*

**Figure 1:**
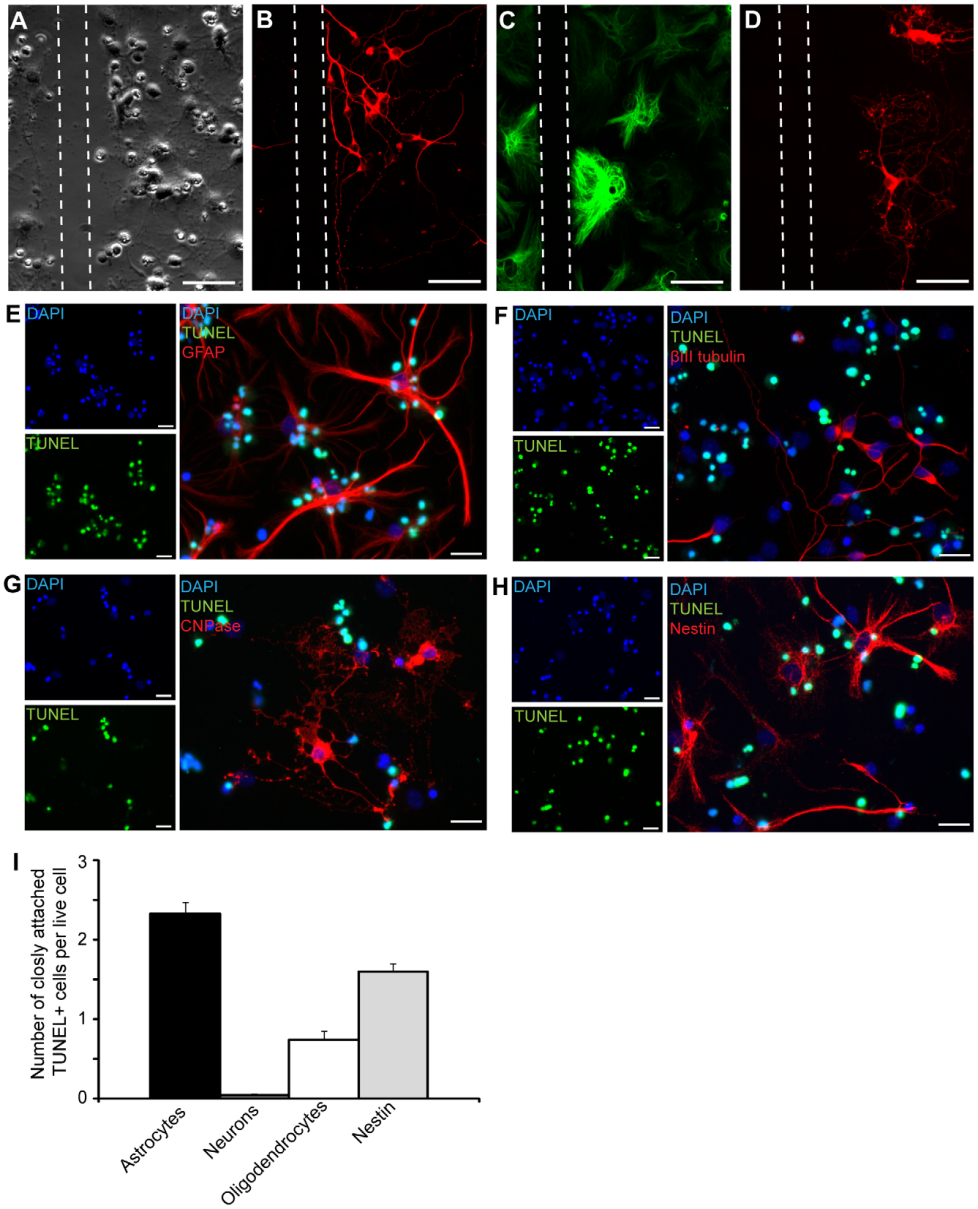
Neurons, astrocytes and oligodendrocytes respond differently to injury *in vitro*. (A) Phase contrast micrograph of a scratch injury to a mixed cell culture of neurons, astrocytes and oligodendrocytes. Injury was induced with a scalpel and 22 hours after the cut the cell cultures were fixed in 4% PFA and stained with specific antibodies against (B) neurons (β III tubulin, red), (C) astrocytes (GFAP, green) and (D) oligodendrocytes (CNPase, red). (E–F) Many TUNEL positive cells (green) with condensed nuclei (blue) were found to be in close contact with (E) GFAP positive astrocytes, but not with (F) neurons and only occasionally with (G) oligodendrocytes. Most of the (H) nestin positive cells were associated with TUNEL positive cells. (I) Quantification of dead, TUNEL positive cells that overlapped with either cell type show that astrocytes are the primary cell to be associated with dead cells. Dashed lines represent the scratch. Scale bars equal 50 µm (A–D) and 20 µm (E–H).

In order to study cellular processes and interactions between neurons and astrocytes following injury we set up an *in vitro* model of brain injury that is suitable for close-up time-lapse experiments. In short, E14 mouse cortical stem cells were differentiated into astrocytes, oligodendrocytes and neurons on pre-coated glass cover slips for 8–10 days in serum-free medium. Neural lacerations were then induced with a scalpel cut 20 times through the mixed cell culture (10 times perpendicularly in either direction, approximately 2 mm apart) (Figure 1A–D). The neurons were notably attracted to the injury and grew along the cut without overstepping the boundaries created by the scalpel on glass (Figure 1B). Similarly to neurons, the astrocytes were found along the laceration (Figure 1C) whereas oligodendrocytes neither migrated towards the laceration nor grew along it, but rather averted the injury site (Figure 1D). In more detailed studies of the cell cultures following scratch injury, we found that many GFAP positive astrocytes were tightly bound to cells with highly condensed nuclei, typical for nonviable cells. We noted that single astrocytes often were in close contact with not only one, but several condensed cells and even repeated washing did not clear the cells. To elucidate the number of dead cells that was co-localized with astrocytes, oligodendrocytes, neurons and nestin-positive cells, injured cultures were labeled with specific antibodies against GFAP, CNPase, βIII tubulin and nestin in combination with TUNEL and DAPI (Figure 1E–H). Quantification of dead, TUNEL positive cells that overlapped with either cell type revealed that astrocytes were closely associated to an average of 2.32±0.15 (mean±SEM) TUNEL positive cells, while viable neurons were not at all overlapping with dead cells (0.05±0.73, mean±SEM) (Figure 1I). Oligodendrocytes were sometimes associated to TUNEL positive cells, but much less frequent than the astrocytes (0.73±0.12, mean±SEM) (Figure 1I). Only TUNEL positive cells that were clearly overlapping with the specific cell markers and were situated in proximity to the viable nucleus were counted. CNPase and βIII tubulin are expressed throughout the oligodendrocytic and neuronal extensions, while GFAP is mostly expressed in the central parts of the astrocyte processes, which may in fact result in an underestimation of TUNEL positive cells associated to astrocytes compared to the other cell types. It is important to note that the astrocytes in the cultures are reactive and therefore express nestin without being immature (Figure S1A–C). A proportion of the neurons also expressed nestin, indicating that these cells are still not fully mature (Figure S1D–F). In contrast to nestin-expressing astrocytes that were in close contact with dead cells, we found that the immature nestin-expressing neurons were not, which explains the lower number of dead cells associated to nestin positive cells (1.6±0.1, mean±SEM) compared to GFAP positive cells (2.32±0.15, mean±SEM) (Figure 1I). The discovery, that primarily the astrocytes are associated with dead cells in the culture after injury, led us to speculate that astrocytes have a role in apoptotic cell clearance.

### Astrocytes engulf whole, dead cells

**Figure 2:**
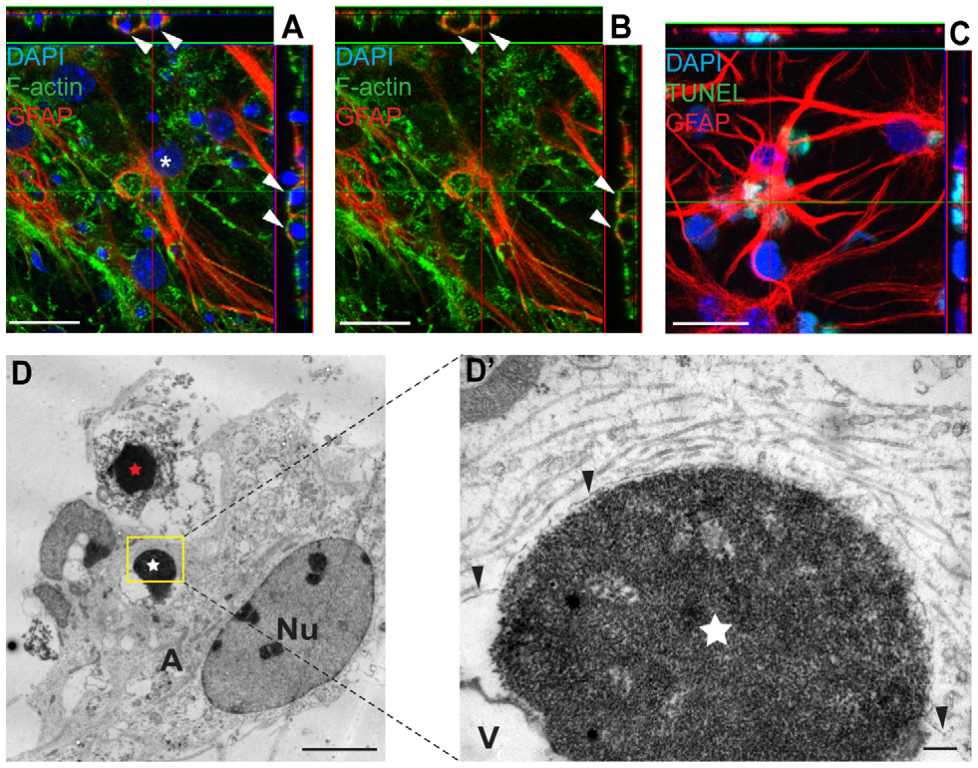
Astrocytes engulf dead cells following neural injury. (A–B) Embryonic astrocytes have engulfed dead cells with highly condensed nuclei. Twenty-two hours after scratch injury, the cultures were fixed and astrocytes were identified with specific antibodies against GFAP. Phalloidin and DAPI labeling was used in order to visualize the actin cytoskeleton and cell nuclei, respectively. Condensed DAPI positive nuclei are present in cytoplasmic vacuoles (white arrows) within the viable astrocyte (white asterisk) and not in direct contact with the cytoskeleton of the astrocyte, indicating a macropinocytotic engulfment mechanism. (C) To prove that the cells with condensed nuclei found within astrocytes are *de facto* dead, we labeled cultures with the apoptotic marker, TUNEL. (D) TEM image of an astrocyte that appear to be in the process of engulfing a dead cell (red star). The dead cell displays the hallmark chromatin traits of apoptosis, but also secondary necrosis as seen by the ruptured cell membrane. The astrocyte contains a previously ingested dead cell (white star) in a spacious vesicle. The astrocyte is marked by an A and the nucleus is denoted Nu. (D') The area marked by a yellow rectangle in D at higher magnification. The ingested dead cell (white star) is contained in a membrane-enclosed compartment (black arrowheads) that is closely linked to the membrane on one side of the dead cell and then diverge to create the vesicle (denoted V) seen in D. Scale bars: 20 µm (A–C), 5 µm (D), 200 nm (D').

To elucidate whether the cells that were in close contact with the astrocytes had actually been engulfed or if they were only attached to the astrocytes we used confocal microscopy and transmission electron microscopy (TEM). Cell cultures studied with confocal microscopy were fixed and labeled with phalloidin for actin visualization, TUNEL to identify apoptotic bodies, DAPI for cell nuclei and specific antibodies against the astrocytic marker GFAP (Figure 2A–C). The gained three dimensional images revealed that the condensed nuclei of the dead cells were located inside the astrocytes, situated in cytoplasmic vacuoles, surrounded by liquid and not in direct contact with the cytoskeleton (Figure 2A–B). This suggests macropinocytotic engulfment, which previously has been shown to be a predominant form of apoptotic cell ingestion. To confirm the non-viability of the astrocyte-garnered cells, TUNEL labeling in combination with GFAP staining was used. This clearly demonstrates that the gathered and engulfed cells are indeed apoptotic (Figure 2C). TEM images in Figure 2D show an astrocyte that appear to be in the process of engulfing a dead cell, as seen by the formation of a phagocytic cup, and another previously phagocytosed dead cell. Although the dead cells displayed characteristics of apoptosis, the rupture of the cell membrane indicates that the cells were going through secondary necrosis (Figure 2D, D'). Furthermore, TEM images confirmed our confocal microscopy findings that the dead, engulfed cell was contained in a vacuolar compartment surrounded by a network of well-organized actin filaments (Figure 2D, D').

### Astrocytes actively collect cell debris

**Figure 3:**
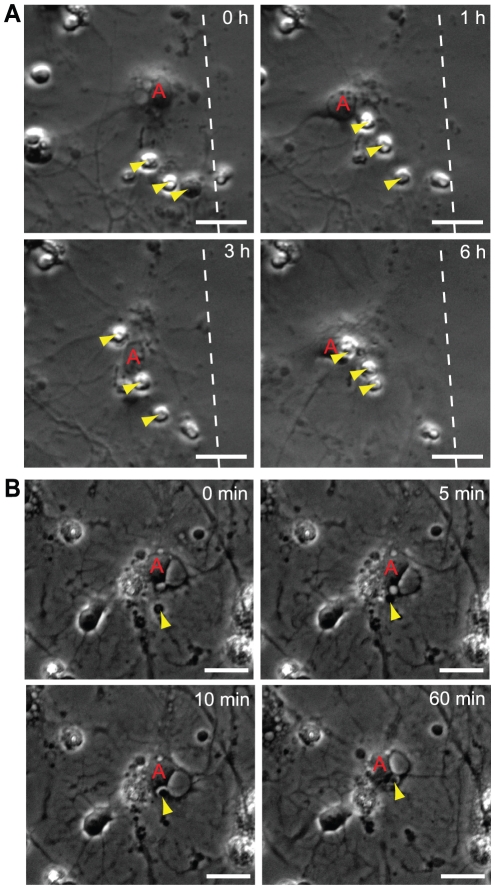
Astrocytes gather cell corpses and engulf cell debris after scratch injury. (A) An astrocyte (red A) gathers dead cells (yellow arrowheads) by adhering to the dead cells with its processes' before dragging the dead cells towards the cell body. First picture is taken 40 minutes after injury and time indicated for each picture is time lapsed after the first image. Scale bar = 20 µm, the dashed lines represent the scratch. (B) An astrocyte (red A) actively gathers and engulfs cell debris (yellow arrowhead) that directly after engulfment appears in a vacuole within the astrocyte. First picture is taken 10 h, 40 minutes after injury and time indicated in the pictures is time lapsed after the first image. Scale bar = 10 µm.

Our studies of cellular events following laceration by time-lapse microscopy revealed that free-floating cell debris and cell corpses, that initially were present around the scratch, effectively were gathered by the astrocytes in the culture (Figure 3A, Video S1). Although fixed astrocytes and neurons can be difficult to distinguish without immunostainings, we could easily identify the cell types in the films based on their phenotype in combination with their specific behavior. Astrocytes had a phenotype of an egg (sunny side up) and were closely attached to the surface of the dish whereas the neurons had round cell bodies and distinct axons. The astrocytes constantly moved their filaments, but did not migrate, in contrast to the neurons, which migrated long distances on top of the astrocyte layer (Video S2 and S3). The time-lapse observations showed that the astrocyte processes' adhered to the dead cells and then started gathering the corpses closer to their cell body (Figure 3A, Video S1). In addition to gathering and engulfing whole, dead cells, astrocytes frequently engulfed and degraded cellular debris as shown in Figure 3B and Video S4.

### Cell corpses induce apoptosis in migrating neurons

**Figure 4:**
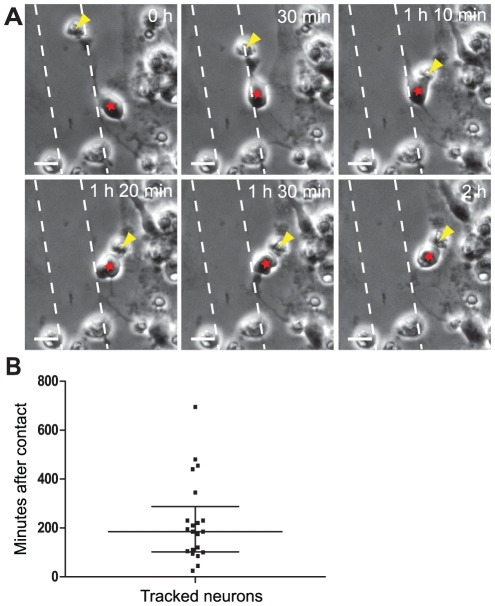
Bystander cell death is induced after direct contact between free-floating cell corpses and healthy, migrating neurons. (A) The cell corpse (yellow arrowhead) is initially connected to the healthy neuron's (red star) axon (0 h), but apoptosis is not induced until the two cell bodies make contact. After the cell body contacts the cell corpse (1 h 10 min, yellow arrowhead), the healthy neuron (red star) rounds up (1 h 20 min), blebs (1 h 30 min) and dies (2 h). First picture is taken 5 h and 50 minutes after injury and time indicated in the pictures is time lapsed after the first image. Scale bars = 10 µm, the dashed lines represent the scratch. (B) To analyze the frequency of bystander cell death, all neurons in 4 different time-lapse films were tracked. In total we found 26 neurons that came in contact with free-floating dead cells. Of these 26 neurons, 21 neurons (80.8%) died within the experiment. The squares represent the survival time for each of these 21 neurons after contact with the dead cell and lines represent the median±interquartile range.

Bystander killing is a phenomenon where dead or dying cells transfer adverse signals to neighboring, healthy cells, either by direct contact or by soluble mediators. We noticed that free-floating cell corpses around the laceration induced bystander cell death of neurons following direct cell-to-cell contact (Figure 4A, Video S4 and S5). Neuronal motility was dramatically enhanced in injured cultures compared to uninjured controls (Video S2 and S3) and since the neurons particularly migrated towards and along the cut, the risk of contact between dead cells and healthy neurons increased after injury (Video S2). Most neurons initially contacted dead cells with their axons, but apoptosis was not induced until the cell body of the healthy neuron was in direct contact with the dead cell (Figure 4A, Video S4 and S5). While bystander killing of neurons was a frequent phenomenon in the injured cell cultures, we never detected contact-induced cell death in glial cells. To analyze the frequency of bystander cell death, we tracked all neurons in 4 different time-lapse films. In total we found 26 neurons that came in contact with free-floating dead cells. Of these 26 neurons, 21 neurons (80.8%) died within the experiment. Most of these 21 neurons (76.2%) die within four hours after the first contact with the dead cell. A group of neurons, however, lived for more than 7 hours, indicating that it might be alternative mechanisms of bystander killing. The survival time for each neuron after contact with the dead cell is shown in Figure 4B. The reason why five of the 26 neurons did not go into apoptosis within the experiment (not included in the graph) is not clear, it is however possible that they would have died if we have continued the time-lapse for some additional hours. To investigate whether the contact-induced apoptosis in neurons could be prevented by inhibition of gap junctions, 100 µM carbenoxolone was added to the cell culture at the time of injury. Treatment with the gap-junction inhibitor did, however, not prevent bystander cell death of neurons in the cultures following injury (data not shown), indicating an alternative mechanism of action.

### Astrocytes protect neurons from bystander cell death

**Figure 5:**
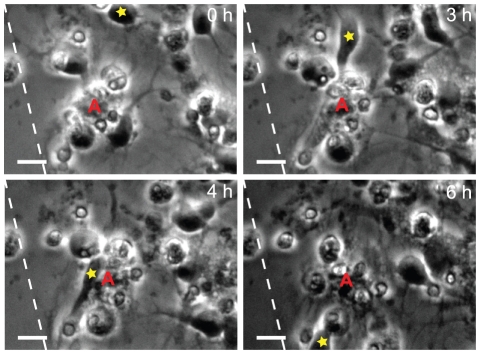
Apoptosis in neurons is prevented by cell corpse removal by the astrocytes. A neuron (yellow star) migrates completely unaffected over an astrocyte (red A) seemingly covered by dead cells. Healthy neurons are protected from bystander cell death by the astrocytes' ability to ingest cell corpses and thereby preventing direct contact that cause apoptosis in neurons. The first picture is taken 10 hours after injury and the time indicated in each picture is time lapsed after the first image. Scale bar = 10 µm, the dashed lines represent the scratch.

The cell death induced by the cut itself and bystander killing of neurons caused a modest (4.3%) increase in the percentage of TUNEL labeled cells in the injured cultures compared to control cultures 22 hours after injury. We hypothesized that the astrocytes have a protective effect on the neurons by taking care of cell corpses in the culture. Our intension was to use specific neuronal markers to confirm our observation from the time-lapse films that primarily neurons die after injury. However, this was not possible due to the loss of filamentous material (including βIII tubulin) during apoptosis. In our time-lapse experiments it was evident that free-floating dead cells imposed a great threat to neuronal survival (Figure 4A–B and Video S4). By tracking neurons in four time-lapse films, we found in total 17 neurons that migrated over dead cells gathered and/or engulfed by astrocytes. None of these neurons died. Whether all dead cells were situated inside the astrocytic cell membrane was indeterminable from the time-lapse films, but it was obvious that the gathering and ingestion of dead cells by the astrocytes had a protective function in regards to the bystander cell death, and thereby important for neuronal survival. The videos of neurons migrating over, what appeared to be cell corpse covered astrocytes, confirmed that although the cells seemed to only be attached to the astrocyte, they were *de facto* ingested and therefore no longer were able to induce apoptosis (Figure 5, Video S4).

### Engulfing astrocytes are present in the brain following acute injury

**Figure 6:**
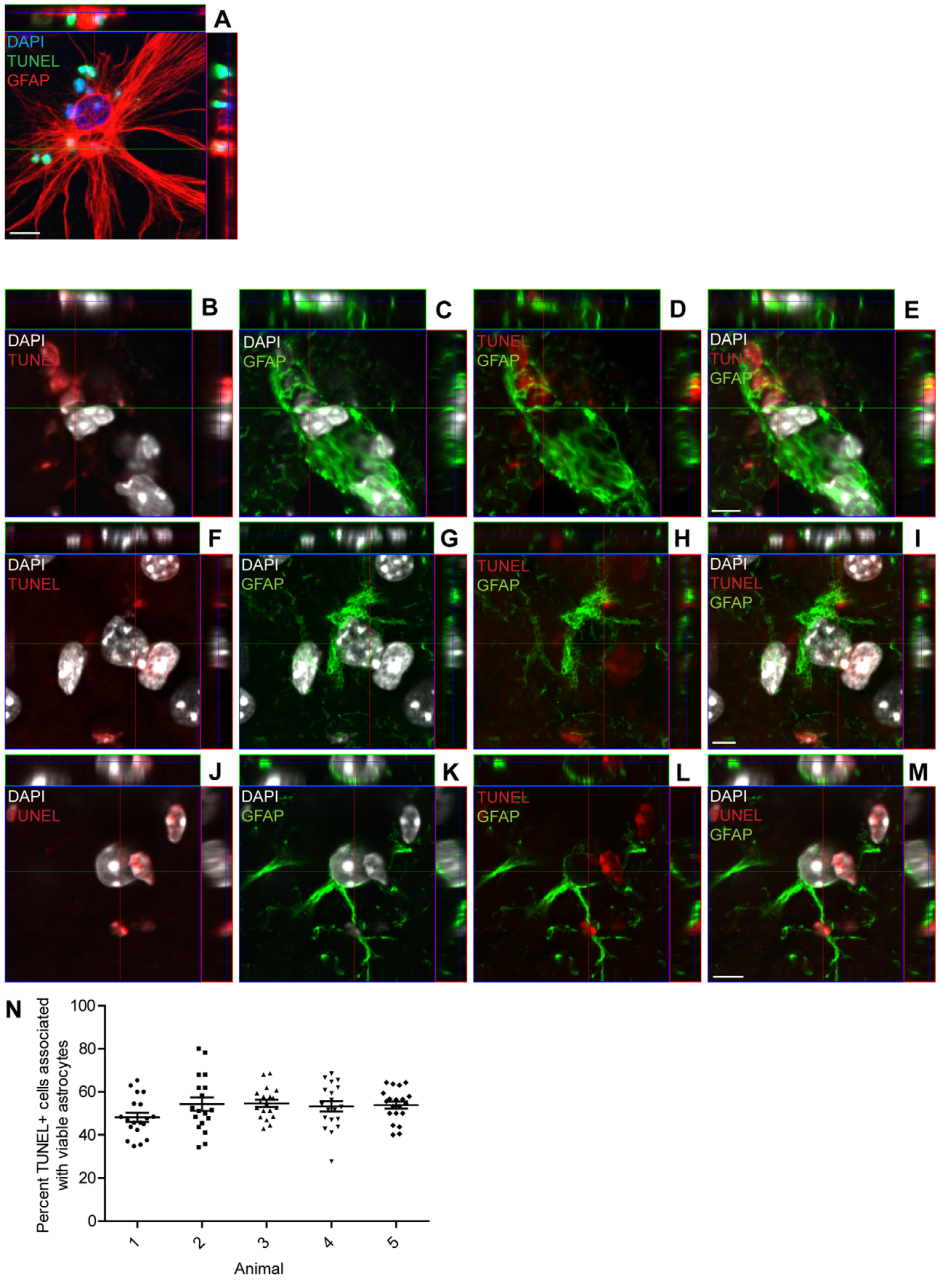
Engulfing astrocytes are present in the brain following acute injury. (A) Astrocytes derived from the adult brain engulf cell corpses. Mixed cell cultures derived from SVZs of adult animals were injured and fixed after 22 hours. Dead cells were identified by TUNEL staining (green), astrocytes with specific antibodies against GFAP (red) and nuclei by DAPI (blue). Confocal micrograph shows a dead, TUNEL positive cell within an astrocyte (white arrows). (B–M) Traumatic brain injury in mice elicits astrocytic engulfment of dead cells at the site of injury. Animals that received CCI-injury were perfused after one (*n* = 5), three (*n* = 5) or seven (*n* = 5) days and cryostat brain sections were labeled with TUNEL (red), DAPI (white) and antibodies against GFAP (green). Confocal micrographs show dead cells within the astrocytes at (B–E) one, (F–I) three and (J–M) seven days post-injury. (N) Scatter plot of the percent TUNEL+ cells associated with viable astrocytes in each animal 7 days post-CCI. For each five animals, two separate sections from bregma levels −1.0 and −2.0 mm, were counted and the percentage in 8–10 fields for each level (40× magnification) were plotted. Scale bars = 5 µm.

To investigate whether the phenomenon of engulfing astrocytes was restricted to cells gained from embryonic tissue, we repeated the experiment with cells derived from the subventricular zone (SVZ) of adult mice. Similar to the embryonic derived astrocytes, the astrocytes from the adult brain also engulfed cell corpses (Figure 6A), indicating that the engulfing astrocytes may have a role in adult tissue. Next, we investigated whether engulfing astrocytes are present *in vivo* following traumatic brain injury. Trauma was induced in adult C57/BL6 mice using a controlled cortical impact (CCI) injury model. One, three or seven days after CCI, the animals were perfused (*n* = 5 per time point) and the brains were sectioned and stained with TUNEL label, DAPI and antibodies against GFAP. Confocal microscopy analysis showed engulfed, TUNEL+ nuclei within the astrocytes in the border zone of the impact site at all time-points (Figure 6B–M), indicating that astrocytes may have a role in clearing cell corpses following acute brain trauma. Notably, we only found TUNEL positive cells within reactive astrocytes with a high expression of GFAP. We found that the presence of engulfing astrocytes was most conspicuous at day 7, most likely because the astrocytes are fully activated by this time. To get an indication of the possible involvement of astrocytes in cell clearance, we quantified the number of TUNEL positive nuclei that co-localized with GFAP of viable astrocytes and the total number of TUNEL positive cells in brain sections from five mice, sacrificed seven days after TBI. For each animal, two separate sections, from bregma levels −1.0 and −2.0 mm, were analyzed and cells in 8–10 fields for each level (40× magnification) were counted. Five examples of photos used for cell counting are presented in Figure S2A–C, D–F, G–I, J–L and M–O. The result shows that in the five mice an average of 52.9±1.0% (mean±SEM) of the TUNEL positive cells are co-localized with viable astrocytes. A similar estimate was made using the activated microglial/macrophage marker Mac-2. The result showed an average of 33.4±1.2% (mean±SEM) of the dead, TUNEL positive cells were co-localized to the viable cells (Figure S3), supporting the hypothesis that astrocytes may have a role in cell corpse removal following brain injury *in vivo*. The percentage of TUNEL positive cells associated with astrocytes in the individual mice is shown in Figure 6N.

### Possible molecular mechanisms of astrocytic phagocytosis

**Figure 7:**
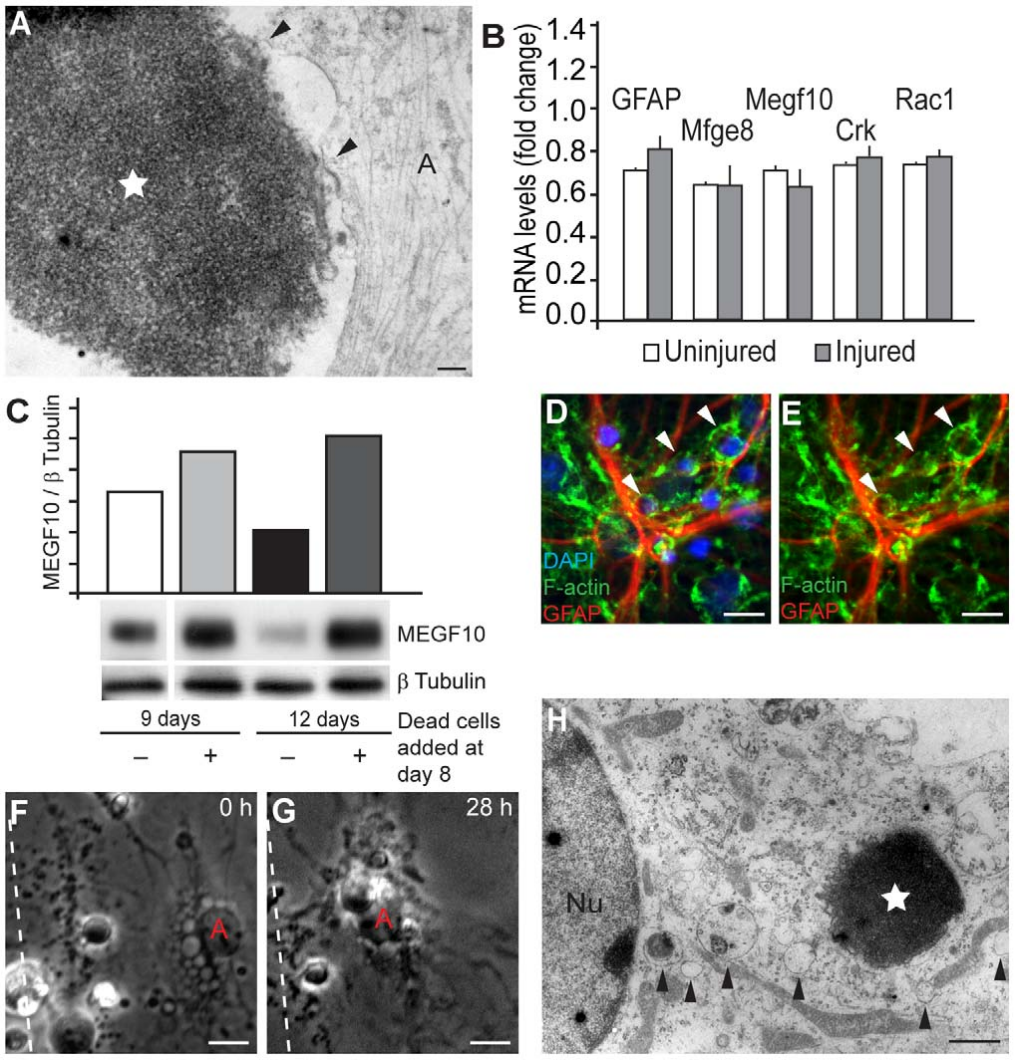
Molecular mechanisms behind the macropinocytosis-like engulfment in astrocytes. (A) TEM image of an astrocyte in the process of engulfing a dead cell (white star) show interaction points (black arrowheads) indicative of receptor/ligand interaction. Dead cells were added to the culture and incubated for 22 h before fixation and prepared for TEM. (B) Phagocytic genes are expressed in the neural cultures. qRT-PCR data showing the expression of *GFAP*, *Megf10*, *Crk*, *Rac1* and *Mfge8*. The average of 5 independent injured and 5 uninjured neural cultures are presented. *Megf10*, *Crk*, *Rac1* and *Megf8* were expressed in all the cultures. Error bars represent SEM. (C) The protein levels of MEGF10 is up-regulated after addition of dead cells. Fresh medium with or without dead cells were added to cell cultures, differentiated for 8 days. The cells were incubated for an additional 1 day (9 days in graph) or 3 days (12 days in graph). Cell lysates were analyzed for MEGF10 expression by Western blot and β Tubulin served as a loading control. (D–E) Engulfed cells are found in macropinocytotic-like vacuoles. Twenty-two hours after scratch injury, the cultures were fixed and astrocytes were identified with specific antibodies to GFAP. Phalloidin and DAPI labeling was used in order to visualize the actin cytoskeleton and cell nuclei, respectively. To be counted as engulfed, the dead cells (condensed nuclei) had to be situated in cytoplasmic vacuoles (white arrowheads) within the astrocytes. (F–H) The highly vacuolized astrocytes appear most active in cell corpse clearing. (F–G) The micrographs show a highly vacuolized astrocyte before engulfment (F) and approximately 28 h later the same astrocyte have ingested several dead cells (G). First picture is taken 25 minutes after injury and time indicated in the pictures is time lapsed after the first image. The dashed line represents the cut. (H) The vacuoles (black arrowheads) can be seen in transmission electron microscope as round vesicles, some apparently empty whereas others contain debris. An astrocyte-ingested dead cell is marked by a white star and the nucleus by Nu. Scale bars: 200 nm (A), 10 µm (D–G), 2 µm (H).

TEM analysis of dead cells in process of being internalized show interaction points between the astrocyte and the cell corps, indicative of possible receptor binding (Figure 7A). The exact mechanisms used in corpse removal by non-professional phagocytes in mammals are still unclear, but many pathways for elimination appear to be highly conserved evolutionary [24]. To find out if the cell cultures express genes of the two most studied pathways of non-professional phagocytosis, qRT-PCR was performed with RNA from 5 independent injured and uninjured cultures. Primers for *Megf10* and *Crk*, the first members in the two parallel *ced* phagocytosis pathways, were designed as well as primers for *Rac1*, in which the two pathways merge. We also included primers for *Mfge8*, an opsonizing agent of apoptotic cells, and *GFAP*. Our qRT-PCR data demonstrate that *Megf10*, *Crk*, *Rac1* and *Mfge8* are all expressed in the cultures, indicating that both pathways might be involved in astrocytic phagocytosis (Figure 7B), although there were no significant differences in expression of the genes in injured cultures compared to uninjured cultures (Figure 7B).

The ortholog to CED1, MEGF10, is a membrane-spanning receptor known to bind to phosphatidylserine (PS) exposed on the surface of apoptotic cells [25]. As previously described, we observed that astrocytes strongly bind dead cells and to investigate if the expression of MEGF10 was increased in the presence of apoptotic cells, we performed Western blot analysis (Figure 7C). Cell cultures, differentiated for 8 days, either received medium with or without apoptotic cells and lysates were prepared after 1 or 3 days incubation. Our results show that there was a clear increase in MEGF10 expression in cultures with dead cells compared to the cultures incubated with medium alone at both time-points (Figure 7C). The average increase, in three separate Western blots experiments, of MEGF10 expression (±SEM) compared to the controls without added cells is shown in Figure S4.

Counting of the engulfed cell corpses in the cultures following injury, revealed that there were in average 1.3±0.11 (*n* = 3, SEM) dead cells inside each astrocyte one day after the scratch injury. Only cells with condensed nuclei that without doubt were situated in a cytoplasmic vacuole within an astrocyte were included (Figure 7D–E). We found that some astrocytes had a very high engulfing capacity, containing several ingested cells, while others contained none. This suggests that there are subclasses of astrocytes within our cell cultures, which is in line with previous findings that have shown both morphological and functional heterogeneity among astrocytes [12], [26]. The more effective astrocytes had a highly vacuolized phenotype (Figure 7F–H), which previously has been shown in fibroblasts to be the consequence of MEGF10 expression [27]. These findings points to the involvement of MEFG10 in the engulfment of cell corpses and debris, although several other pathways may very well be involved.

### Alternative processing of engulfed cell corpses in astrocytes compared to macrophages

**Figure 8:**
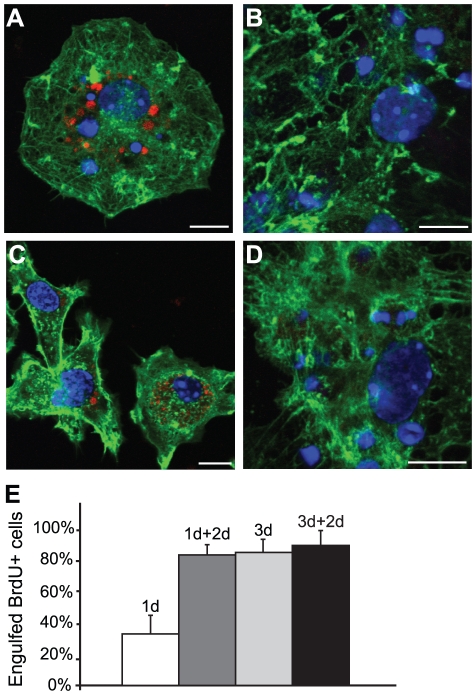
Astrocytes are ineffective in degrading the engulfed cells. Apoptotic cells were pre-labeled with pHrodo-dye and added to (A,C) control macrophage cultures and (B,D) neuronal cell cultures. Parallel neural and macrophage cell cultures were fixed after (A–B) 5 h or (C–D) 3 d. (A) After 5 h, macrophages had already started to degrade phagocytosed cells in the lysosomes (bright red intracellular compartments). (B,D) Astrocytes did not contain red fluorescing material at any time point, indicating that the engulfed cell corpses did not fuse with lysosomes. (C) Dead cells ingested by macrophages had been degraded after 3 days and only remnant pHrodo-dye was apparent intracellularly. (D) In contrast to the macrophages, astrocytes had accumulated more intact DAPI labeled nuclei at day 3, but did not fluoresces red, indicating that the ingested cells had not fused with lysosomes at this time. (E) BrdU labeled, apoptotic cells were added to neuronal cultures and parallel cultures were fixed after 1 and 3 days (1 d respective 3 d in graph) or were carefully washed after 1 or 3 days and incubated for an additional 2 days in medium without apoptotic cells (1 d+2 d respective 3 d+2 d in graph) prior to fixation. Counting of the ingested BrdU+ nuclei show that astrocytes continued to accumulate cell corpses during the 1 day to 3 days of incubation, but after 2 days in medium without dead cells the ingested dead cells had still not been degraded. Error bars represent SEM.

We next sought to investigate what happens with the ingested dead cells in the astrocytes following engulfment. For this purpose we used pHrodo, a novel dye that is nonfluorescent at neutral pH and fluoresces bright red in acidic environments as in the lysosomes. Apoptosis of neural cells was induced by exposing the cells to 480 mJ of UV-light. The apoptotic cells were then labeled with the pHrodo-dye and added to the differentiated neuronal cell cultures and control macrophage cultures. Parallel neural and macrophage cell cultures were fixed after 5 hours and 3 days (Figure 8A–D). In contrast to the macrophages, which already had phagocytosed cell corpses after 5 hours (Figure 8A) (indicated by the brightly fluorescent red intracellular compartments), the astrocytes did not contain red fluorescing material at any time point (Figure 8B,D). Astrocytes still contained intact DAPI labeled nuclei at day 3 (Figure 8D), while the macrophages had degraded the ingested material, as shown by the loss of condensed DAPI stained nuclei (Figure 8C). These results show that dead cells engulfed by astrocytes are not degraded via lysosomic pathways (at least not within 3 days). To further elucidate the capacity of astrocytes to engulf and break down cell corpses, we performed another set of experiments where BrdU labeled, apoptotic cells were added to neural cultures. The reason for labeling the cells with BrdU was to ensure that all cell corpses analyzed in the experiment were from the added, UV-treated cells and had not been ingested prior to the experiment. Parallel cultures were fixed after 1 and 3 days following addition of UV-treated cells or carefully washed after 1 or 3 days and incubated for an additional 2 days in medium without apoptotic cells. By counting the ingested BrdU+ nuclei, we revealed that astrocytes continued to accumulate cell corpses during the 1 day to 3 days of incubation, but after 2 days in medium without dead cells the ingested dead cells had still not been degraded (Figure 8E). After 1 day, many of the dead, BrdU+ cells were attached to the astrocytes and 35.4±2.8% (*n* = 3, SEM) of the BrdU+ cells were found inside the astrocytes. The percentage of engulfed BrdU+ cells in cultures washed after 1 day and cultured in medium alone for 2 extra days had increased to 70.5±2.6% (*n* = 3, SEM), demonstrating that most of the BrdU+ cells were attached to the astrocytes at day 1, but required additional time for engulfment. After 3 days incubation in medium with dead cells, 72.0±2.3% (*n* = 3, SEM) of the BrdU+ cells in the culture had been engulfed by astrocytes and in cultures incubated for 3 days with dead cells and 2 additional days in medium alone, the percentage of ingested BrdU+ cells were 78.4±2.3% (*n* = 3, SEM). These results show that astrocytes, although being effective in engulfing dead cells, are very inefficient in breaking down the ingested material compared to macrophages.

## Discussion

While professional phagocytes have been extensively studied in various contexts, little is known about the biological significance and the molecular mechanisms of non-professional phagocytes. We demonstrate that astrocytes, the most numerous cell type within the CNS, engulf dead cells following neural injury. Our fundamental findings are that astrocytes effectively gather and engulf cell corpses following injury in a mixed neuronal culture and thereby rescue migrating neurons from bystander cell death. Moreover, we show that astrocytes containing multiple TUNEL positive nuclei are present at the lesion site in mice after TBI, indicating a possible role for astrocytes in engulfment of apoptotic cells following acute brain injury *in vivo*.

There are two previous *in vitro* studies indicating that astrocytes are capable of engulfing whole cells [14], [15]. In these studies hematoxilin staining was used to demonstrate the presence of multinuclear cells, but whether the cells were indeed ingested and not merely attached to the astrocytes were not clarified [14], [15].

In the present study we present important data about the mechanism and function of astrocytic engulfment. By using TEM and confocal microscopy analysis we demonstrate that apoptotic cells are indeed ingested by the astrocytes following injury *in vitro*. This is the first unambiguous evidence that astrocytes are able to engulf whole, dead cells. Furthermore, our time lapse films show that the engulfing astrocytes protect neurons from bystander killing by ingesting the dead cells and thereby preventing the cell corpse contact that would otherwise initiate apoptosis in the neuron.

Naturally occurring bystander killing of neurons has been demonstrated in the developing retina were the gap-junction inhibitors carbenoxolone or octanol reduced, but not abolished, the bystander killing [28]. Addition of carbenoxolone did not prevent contact-induced cell death of neurons in our *in vitro* model of neural injury, supporting the hypothesis that gap-junctions are not the only mechanisms for bystander cell death of neurons. An alternative mechanism might be binding of the necrotic/apoptotic cell to death receptors, such as Fas and tumor necrosis factor receptor-1, on the healthy cell [29]. By tracking the neurons in the time-lapse films we show that most neurons die within four hours following cell corpse contact. Some neurons, however, live much longer than that indicating that there may be alternative mechanisms of bystander cell death in the cultures. Interestingly, it has been suggested that astrocytes, in contrast to neurons, are resistant to apoptosis induced by Fas-ligand [30], which could explain why the bystander effect was only seen in neurons and not in glia. There is evidence that cells individually exposed to radiation induce bystander apoptosis of unirradiated cells by the release of toxic signals to the extracellular fluid rather than through gap-junctions [31]. It is, however, clear from our time-lapse films that the induction of cell death in our culture system requires a direct contact between the cell corpse and the cell body of the healthy neuron. Migration of the neuron close to a dead cell or contact via an axon is not sufficient to induce cell death, confirming that the mechanism for bystander killing is mediated via contact of cell membrane components on both cells and not by diffusing extracellular factors.

The exposure of PS on the cell surface of apoptotic cells has been described as an almost universal recognition ligand for professional phagocytes. Recently, it has however been demonstrated that that macrophage engulfment of cells that had undergone necrotic programmed cell death also is PS-dependent [32]. The morphology of the dead cells seen inside astrocytes in our TEM images showed the characteristics of apoptosis, but there were also evidence pointing to secondary necrosis. Furthermore, there were specific interaction points between the astrocyte and the cell corpse, indicating that the internalization of cell corpses is dependent on receptor/ligand interactions. Whether the engulfment of the cell corpses by astrocytes is dependent on PS-exposure or not was not elucidated in this study, but it has previously been suggested that the occurrence of PS on the surface of apoptotic cells is patchy and highly localized, similar to what we see in our TEM images [24].

Insight into the molecular mechanisms by which nonprofessional tissue-resident cells affect apoptotic clearance initially came from *Caenorhabditis elegans*, which lacks professional phagocytes. Two evolutionary conserved signaling pathways of recognition and internalization of dead cells have been identified in the nematode [33], [34], [35]. The first mammalian homologous pathway consists of Crk, Dock180 and Elmo and the other consists of Megf10, Gulp 1 and Abca1. The pathways converge at Rac1 which is involved in the cytoskeleton rearrangement required for engulfment [33]. A gene profiling study shows that molecular components of both engulfment pathways are expressed at high levels in acutely isolated astrocytes from postnatal and adult mouse forebrain, suggesting an important role for developing and mature astrocytes in the clearing of dead cells. Furthermore, the authors show that *Mfge8* was one of the few highly expressed genes in astrocytes [36]. Another study shows, by *in situ* RNA hybridization and qRT-PCR, that *Mfge8* is primary expressed by a subset of astrocytes in the CNS and that *Mfge8* deficiency resulted in accelerated prion pathology and an elevated number of apoptotic cerebellar granule cells [37]. This suggests that MFGE8 is required for efficient removal of apoptotic cells in the CNS and possibly also for degradation of prions. Our qRT-PCR data show that *Megf10* and *Crk* are both expressed in the neural cultures as well as *Rac1* and *Mfge8*. Moreover, we show that the protein expression of MEGF10 is increased in the presence of apoptotic cells. Although our qRT-PCR and Western blot data support that astrocytic engulfment is receptor-mediated, the transmission electron analysis strongly suggest that the astrocytes ingest dead cells by a macropinocytosis-like mechanism (the engulfed cell corpses are situated within vacuoles that have characteristics of macropinosomes). Hence, the exact engulfment and degradation pathway is still uncertain. Recent data from other research groups indicate that the distinction between various engulfment mechanisms is not as clear-cut as previously presumed and there may be intermediary mechanisms of receptor mediated engulfment and macropinocytosis [38]. Our TEM images show that both apoptotic and secondary necrotic cells are ingested by the astrocytes, which supports the hypothesis that several mechanisms of engulfment could be involved in the clearing of dead cells. Interestingly, in our comparison of phagocytosis by astrocytes and macrophages we found that astrocytes, similar to macrophages, engulf dead cells, but do not effectively degrade the material in contrast to the macrophages. By using the pHrodo-labeling technique we demonstrate that cell corpses ingested by macrophages fuse with lysosomes whereas dead cells engulfed by astrocytes do not (at least not within 3 days). This finding, that astrocytes effectively engulf dead cells, but then store the material rather than degrade it, probably explains why multinuclear astrocytes are found in the brain tissue of patients with various disorders including MS, Creutzfelt-Jacob disease and stroke [8], [9], [10], [11], [12], [13].

In an *in vivo* model of excitotoxicity in rats it was noted that reactive astrocytes do not generally show TUNEL positive nuclei, but were frequently surrounding TUNEL positive nuclei with their projections [39]. By confocal microscopy analysis of tissue sections we demonstrate that reactive astrocytes, containing several TUNEL-positive cells, are present by the border zone of the lesion site at one, three and seven days after TBI in mouse. Moreover, cell quantifications demonstrate that 52.9±1.0% (mean±SEM) of the TUNEL positive cells are co-localized with GFAP in viable astrocytes seven days post-TBI. This result indicates that astrocytes behave similarly *in vivo* as in our *in vitro* injury model and that neighboring astrocytes may partake in the clearing of cell corpses following acute brain trauma. We also showed that activated microglia or macrophages at the lesion site were co-localized with apoptotic cells at a lower ratio than the astrocytes (33.4%±1.2%, mean±SEM) at seven days post injury. Notably, Mac-2 is a cell surface located protein whereas GFAP is expressed in the central part of the astrocyte. TUNEL positive cells that are co-localized with GFAP are therefore most likely inside the astrocytes (as verified in the confocal micrographs), but do not necessary reflect engulfment since the dead cells may be situated outside the plasma membrane.

Since the professional phagocytes are faster at degrading the ingested material according to our *in vitro* data, but are much fewer than the astrocytes, it is hard to accurately estimate the respective cell types' significance in the clearing of dead cells *in vivo*. However, one can speculate that the astrocytes situated in the injured tissue might be important for cell survival directly after the insult, before recruitment of professional phagocytes has taken place or if there is an overload of dead cells for the professional phagocytes to handle and later when the inflammatory response has declined. It is known from several studies that brain tissue damage following TBI is a chronic process with ongoing secondary brain tissue atrophy for several years after the primary injury [40]. Very little is known about the inflammation at later time points, since most TBI studies concentrate on the first weeks following injury. It has, however, been reported that both reactive astrocytes and macrophages/microglia are still present in the injured brain one year following trauma in rats [41].

It is known that phagocytosis in mammals not only serves as an effector mechanism to clear infectious agents, dead cells or unwanted materials, but recognition and internalization by phagocytes also involves release of chemotactic factors [42]. It is possible that the engulfing astrocytes situated within or close to the injured tissue causes subsequent recruitment of cells to the injury site by releasing chemoattractants.

A high survival rate of the neurons adjacent to the acutely damaged brain tissue is extremely important for the outcome following brain trauma. An effective and instant clearance of dead cells is most likely central in saving the undamaged neurons from secondary cell death due to the release of toxic substances and bystander killing. In order to develop therapeutic strategies to increase cell survival in the damaged brain, a better understanding of the cellular and molecular mechanisms of apoptotic clearance is highly desirable. In this study we have focused on the role of engulfing astrocytes following physical injury, but we consider it probable that this process could be important in many other brain conditions.

## Materials and Methods

### Animals

All animal experiments were in line with the Swedish animal welfare legislation and the study was specifically approved by Uppsala Animal Ethics Committee, Uppsala, Sweden (Permit number: C 234/8) before the study was started. The mice were housed at 24°C in 12 h light/dark cycles with access to food and water *ad libitum*. The pregnant mice were kept in separate cages for no more than one week upon arrival, whereas the mice used in the *in vivo* study, were kept 4 mice per cage for the duration of the experiments. Cortices from C57/BL6 E14 mouse embryos and adult tissue from male C57/BL6 (6–8 weeks) were used in the *in vitro* part of this study and adult C57/BL6 mice (20–25 g) were used for the *in vivo* experiments.

### Neural cell cultures

Dissected cortices from E14 mice were dissociated in GIBCO Hank's Balanced Salt Solution (1×) supplemented with 8 mM HEPES buffer solution and 50 units of penicillin and 50 µg of streptomycin per ml (Invitrogen), hereafter only referred to as HBSS. The cell suspension was centrifuged and resolved in medium (GIBCO Dulbecco's Modified Eagle Medium (D-MEM/F12) with GlutaMAX (×1) supplemented with 50 units/ml^−1^ penicillin and 50 µg/ml^−1^ streptomycin, 8 mM HEPES buffer, 1×B-27 serum free supplement) fortified with 10 ng ml^−1^ Fibroblast Growth Factor 2 (FGF2) (Invitrogen) and 20 ng ml^−1^ natural mouse Epidermal Growth Factor (EGF) (Becton Dickinson). The cells were grown non-adherently into neurospheres and passaged every 3^rd^ to 5^th^ day by dissociation in HBSS and resuspension in new medium supplemented with mitogens at concentrations previously described. Prior to experiment, cells were dissociated in HBSS and plated as a monolayer on coverslips coated with poly-L-ornithine (Sigma-Aldrich Inc.) and laminin (Invitrogen). The first 24–48 h cells were maintained in EGF and FGF2 supplemented medium and thereafter replaced with mitogen-free medium to initiate cell differentiation. The medium was replaced in full every second to third day during the differentiation period of 8–11 days. For studies of adult neural cultures, neural stem cells were isolated by dissection of the forebrain sub-ependyma of adult mice in HBSS. The tissue was digested with NeuroCult® Enzymatic Dissociation Kit for Adult CNS Tissue (Stem Cell Technologies) and the cells were cultured as the embryonic cells described above, but only passaged once a week. For both embryonic and adult cells, neurospheres from passage 1–3 were used for experiments.

### Macrophage cell cultures

Spleens from C57/BL6 mice were harvested and mashed in 3 ml HBSS using a syringe plunger. The cell suspension was centrifuged, and the pellet was suspended in 1 ml of sterile red blood cell lysis buffer (2.075 g ammonium chloride, 0.25 g of potassium bicarbonate and 25 µl of 0.5 M EDTA in 250 ml distilled water). Following incubation in RT for 5 min the cells were centrifuged and the supernatant carefully removed. The cells were dissolved in DMEM with 10% fetal bovine serum (Invitrogen) and seeded on poly-L-ornithine coated glass coverslips (approximately 6 glasses/spleen). The plates were incubated in 37°C, 5% CO_2_, humidified air for 2 h followed by washing of the non-adherent cells twice and administration of new medium. The macrophages were cultured for 2 days prior to experiment.

### In vitro injury model

The injury was induced with a scalpel cut 20 times through the differentiated neural culture, 10 times perpendicularly in either direction, approximately 2 mm apart. Injured cultures were either used for time-lapse experiments immediately after the cut or kept for immunostaining experiments. Cultures used for immunostaining experiments were incubated for 18–24 h or 32 days before fixation in room temperature (RT) for 10–20 min in 4% paraformaldehyde (PFA) (Sigma-Aldrich Inc.) in PBS. For time-lapse experiments with gap-junction inhibitor, 100 µM carbenoxolone was added to the cell culture at the time of injury.

### Traumatic brain injury

Controlled cortical impact (CCI) was performed as previously described [43], [44] after isoflurane (4% in air) induced anesthesia for approximately 2 min. The mice were moved to a stereotaxic frame and anesthesia was maintained by 1.2% isoflurane in mixture with 70% nitrous oxide and 30% oxide through a nose cone. Core body temperature was controlled at 37±0.3°C by a heating pad connected by a rectal probe (CMA150, CMA, Stockholm, Sweden). A midline incision was made after an s.c. injection of bupivacaine (Marcain®, AstraZeneca, Sweden). A craniotomy, 4-mm-diameter, was created 1 mm posterior to bregma over the right parietal cortex extended laterally towards the temporal crista. The stereotaxic frame was moved to the CCI-device (VCU Biomedical Engineering Facility, Richmond, Virginia, USA) with a 2.5 mm diameter piston. The injury was produced by a 0.5 mm compression of the brain at a speed of 3 m/s and the wound was closed up with interrupted sutures. Animals were sacrificed 1 day (*n* = 5), 3 days (*n* = 5) and 7 days (*n* = 5) post injury by a pentobarbital sodium (600 mg/kg) overdose and transcardial perfusion with PBS followed by 4% phosphate-buffered formaldehyde (Histolab AB, Gothenburg, Sweden) and two naïve animals served as controls.

### pHrodo succinimidyl ester (pHrodo)

The dye reacts with amine groups on the cell surface and is non-fluorescent at neutral pH, but if phagocytosed and fused with lysosomes the emission of red light increases as the pH lowers. Neurospheres were separated as previously specified and cells killed by 480 mJ of UV light in a UV Chamber (GS Gene linker, BioRad). The cells were thereafter pre-stained as previously described [45] with pHrodo dye in medium for 45 min, in RT. The cells were washed once with HBSS and solved in medium before added to neural cell cultures or macrophages (approximately 6×10^5^ cells ml^−1^).

### Bromodeoxyuridine (BrdU) labeling

Neurospheres were grown in the presence of Cell Proliferation Labeling Reagent for immunocytochemistry (GE Healthcare) diluted 1∶1000 in cell medium supplemented with EGF and FGF2. The pre-labeled cells were dissociated as previously described, solved in 4 ml medium and added to a 10 cm in diameter Petri dish. Apoptosis was induced by UV light as described above and the cells were incubated with differentiated neural cultures for 1 day and 3 days before fixation in 4% PFA. Parallel cultures were carefully washed after 1 or 3 days and incubated for an additional 2 days in medium alone prior to fixation. Nuclease/anti-5-bromo-2′deoxyuridine (1∶100; Amersham Cell Profileration Kit, GE Healthcare) were added to coverslips and incubated for 1 h at room temperature. Coverslips were washed three times in PBS and incubated with secondary antibody (1∶200; anti-mouse Cy3) for 1 h, before washed three times in PBS and incubated for 1 h with Phalloidin-FITC (1 µM in PBS, Sigma-Aldrich). After three washes in PBS the coverslips were mounted on glass slides with Vechtashield containing DAPI (Vector).

### Immunostaining and other staining techniques

Primary antibodies used in this study included: Tubulin beta III isoform (βIII tubulin, 1∶200, CHEMICON International) produced in mouse, rabbit antibody to Glial Fibrillary Acidic Protein (GFAP, 1∶400, DakoCytomation), 2′,3′-cyclic nucleotide 3′-phosphodiesterase (CNPase, 1∶500, Sigma) produced in mouse, nestin (1∶200, Abcam) produced in mouse or rabbit and Mac-2 (1∶200, Cedarlane) produced in rat. Secondary antibodies (IgG) used were: AlexaFluor 488-conjugated antibody to rabbit or mouse (1∶200, Molecular Probes), Cy3-conjugated mouse or rabbit antibody (Sigma-Aldrich) and Cy3-conjugated rat antibody (1∶200, Invitrogen). Alternative staining techniques included apoptotic detection by TUNEL staining (Roche Applied Science) and F-actin staining by Phalloidin-FITC (1 µM in PBS, Sigma-Aldrich). Cell culture coverslips were permeabilized and blocked for 30 min in 0.1% Triton X-100 (vol/vol, Sigma) and 5% natural goat serum (NGS) (vol/vol) in PBS. Incubation of primary antibodies was performed in either RT for 1–4 h or overnight in 4°C. Coverslips were washed three times in PBS before incubation with secondary antibody for 1 h in 37°C. The samples were washed three times before mounted with Vectashield Hard Set mounting medium, with or without 4′,6-diamidino-2-phenylinodole (DAPI, Vector). TUNEL-staining was performed, after permeabilization, as instructed by the manufacturer (Roche Applied Science). Brains were cryostate-sectioned coronally at a thickness of 14 µm. Permeabilization and blocking was performed for 1 h in RT in 0.3% Triton X-100 in PBS (0.3% Triton/PBS) and 5% NGS. Primary antibody (1° ab) was diluted in 0.3% Triton/PSB with 0.5% NGS and incubated O/N in 4°C. Sections were thoroughly washed in 0.3% Triton/PBS and incubated on shaker in RT with secondary antibody (2° ab) for 2 h and washed in 0.3% Triton/PBS. The sections were mounted on glass with Vectashield containing DAPI (Vector).

### Transmission Electron Microscopy

Cells were cultured as previously described, but seeded directly on the plastic surface. Apoptosis was induced by UV light as described above and the dead cells were incubated with differentiated neural cultures for 1 day and 3 days before fixation. Prior to fixation in 2.5% glutaraldehyde in sodium caccodylate buffer (SCB) the cell cultures were washed once in PBS. The fixation was left overnight in 4°C and the cultures were then rinsed with SCB for 10 min. The dishes were incubated in 1% osmium tetroxide in SCB at RT for 1 h followed by a 10 min in SCB. Dehydration was performed with 70% ethanol for 30 min, followed by 95% ethanol for 30 min and 99.7% ethanol for 1 h. The dishes were then rinsed with a little plastic and a new, thin layer of plastic was added to the cells for 2–4 h to permit evaporation of the alcohol. Thereafter, a second plastic layer was poured and left overnight before a thicker, newly made plastic layer was added. The dishes were incubated in RT for 1 h before polymerization in oven (60°C) for 48 h. Plastic capsules attached to the plastic were used as handles during sectioning. The sectioning was carried out at a slight angle to increase the chance of finding engulfed material. Sections were studied in a Hitachi H-7100 transmission electron microscope.

### Quantitative Reverse Transcriptase PCR

Cell cultures from cortices were used as previously described, with the exception of severity of injury, which was made by 20 cuts in either direction opposed to the usual 10. After overnight incubation after injury, cultures were washed with PBS and total RNA extracted using RNAeasy Mini Kit (Qiagen). RNA concentration (ng/µl) was normalized from the absorbance at 260 and 280 nm, detected by NanoDrop ND-1000 spectrophotometer (NanoDrop Technologies). The following primers used all spun exon-intron boundaries (GeneBank accession number, upper and lower primers are stated): *Gfap* (NM_010277, 5′-CGG GAG TCG GCC AGT TAC CAG-3′ and 5′-TTT CCT GTA GGT GGC GAT CTC-3′), *Megf10* (NM_001001979, 5′-CCA GCC AAC AGG AAT GTC TAT-3′ and 5′-ACT GGC AGC AGG TCA TAA TG-3′), *Rac1* (NM_009007, 5′-CCG CAG ACA GAC GTG TTC T-3′ and 5′TGT CGC ACT TCA GGA TAC CA-3′), *Crk* (NM_133656, 5′-CCA TTT ATG CCA GGG TTA TC-3′ and 5′-GTG ACC TCG TTT GCC ATT AC-3′), *Mfge8* (NM_008594, 5′-ACC TAG CCT CCC GTT GTT CT-3′ and 5′-ACG ATC CCT GTG CGG TAC A-3′). Ten ng of total RNA sample was added to each microwell and all samples carried out in duplicates and analyzed during the same run. Fragments (approximately 100 base pairs long) were amplified by the Bio-Rad iScript One-Step RT-PCR Kit with SYBR Green (Bio-Rad Laboratories) during 36 cycles using a MyIQ thermal cycler (Bio-Rad). Reverse transcription was performed at 50°C for 10 min and verification of the PCR-products specificity was assessed by measuring melting curves by increasing the temperature from 55.0 to 94.5°C in 0.5°C increments. An average threshold value (ΔCt) was calculated from each of the five independent cultures and the corresponding ΔCt of the injured culture was subtracted. The resulting differences were converted to linear increase with the corresponding uninjured culture as reference. The average increase and standard error of mean for each primer was calculated from the five injured and uninjured cultures.

### Western blot analysis

Protein lysates were prepared by incubating the cells in lysis buffer (20 mM Tris pH 7.5, 0.5% Triton-X-100, 0.5% Deoxycholic acid, 150 mM NaCl, 10 mM EDTA, 30 mM NaPyroP) supplemented with 15% protease inhibitor cocktail (Roche) and sodium orthovanadate (Na_3_VO_4_) (0,1 M, Sigma) on ice for 30 min prior to 30 min centrifugation at 12000× g at 4°C. 30 µg protein was loaded on a SDS-PAGE gel (NuPAGE 4–12% Bis Tris Gel, Invitrogen) and the gel was run at 200 V for 90 min in NuPAGE MES buffer (Invitrogen). The proteins were blotted using a PVDF filter (0.2 µm, Invitrogen) pretreated in methanol for 5 min and for 10 min in transfer buffer (Invitrogen). Transferring was done at 25 V, 120 mA for 100 min. The filter was blocked in 5% BSA in 0.2% Tween in PBS (PBS-T) for 60 min and then washed 2 times in PBS-T prior to incubation with primary antibody in 0.5% BSA and 0.2% PBS-T at 4°C over night. Primary antibodies were: anti-MEGF10 (Sigma-Aldrich), diluted 1∶250 and anti-β Tubulin (Imgenex) diluted to 0.25 µg/ml. After washing, the filter was incubated with peroxidase-conjugated secondary antibody for 1 h at RT, washed in PBS-T and developed using enhanced chemiluminescence (ECL) system (GE Healthcare). Before rehybridization the filter was dehybridized in 0.4 M NaOH at RT for 10 min and washed 4×5 min in PBS-T.

### Cell Counting and Statistics

#### Immunostainings - cell cultures

For the TUNEL data, positive cells in 7–10 fields/cover slip were counted in five independent experiments. The ratio between live cells (TUNEL−, DAPI+) and dead (TUNEL+, condensed DAPI+) cells were calculated and the difference between injured and uninjured cultures was given as percent increase. To estimate the number of engulfed cell corpses per astrocyte, the total number of ingested cells and the total number of astrocytes were counted in 10 fields per experiment (*n* = 3). Only cells with condensed nuclei that without doubt were situated in a cytoplasmic vacuole within an astrocyte were considered to be ingested cells. For the BrdU-experiments 10 fields/time point per experiment (*n* = 3) were micrographed and the total number of astrocytes (GFAP+), total number of BrdU+ cells and the number of ingested BrdU+ cells (in a vacuolized phalloidin positive compartment) were counted. A ratio between ingested cells over numbers of astrocytes was given ± SEM.

#### Immunostainings - tissue sections

 For each five animals, two separate sections from bregma levels −1.0 and −2.0 m.m., were analyzed and cells in 8–10 fields for each level (40× magnification) were counted. The total number of double positive (DAPI and TUNEL) dead cells and the number of dead cells that was closely associated/bound to viable, astrocyte cell nuclei (GFAP+) was counted. The results are presented as percent (±SEM) dead cells associated with viable astrocytes.

#### qRT-PCR and western blot

Statistics of the qRT-PCR data (see Quantitative Reverse Transcriptase PCR) was calculated using the non-parametric Mann-Whitney test in Statistica (StatSoft).

#### Time-lapse experiments

Four videos were analyzed frame by frame (5 min between individual images). Single migrating neurons were followed and the time a neuron contacted a rounded, free-floating dead cell was recorded. The same neuron was then continuously followed and the time of death was noted and the time between contact and death was calculated. Death was defined as the time when the neurons had rounded up and lost its extensions by blebbing. The distribution of the time when the total number of neurons died was analyzed for normalcy using Shapiro-Wilk normality test (GraphPad Prism). The time points are presented as the median±interquartile range, due to the failure to meet normalcy. Neurons that were migrating over and/or through groups of dead cells garnered by the astrocytes were also followed to elucidate whether they died and presented as the total number of cells that died.

## Supporting Information

Figure S1 **Astrocytes and immature neurons express nestin.** Mixed cell cultures of neurons, astrocytes and oligodendrocytes were fixed in 4% PFA and stained with specific antibodies against nestin and (A–C) GFAP, (D–F) β III tubulin, and (G–I) CNPase.
(TIF)

Figure S2 **Examples of photos used for quantification of TUNEL and GFAP co-localization** ***in vivo*** **.** Trauma was induced in five mice using a controlled cortical impact (CCI) injury model. Seven days post-injury, the animals were perfused and the brains were sectioned and stained with TUNEL label, DAPI and antibodies against GFAP. Five representative fields, A–C, D–F, G–I, J–L and M–O are shown.
(TIF)

Figure S3 **Quantification of TUNEL+ cells co-localized with Mac-2 positive cells** ***in vivo*** **.** Trauma was induced in five mice using a controlled cortical impact (CCI) injury model. Seven days post-injury, the animals were perfused and the brains were sectioned and stained with TUNEL label, DAPI and antibodies against Mac-2. Ten representative fields per animal (*n = 5*) were counted and the percent TUNEL+ cells associated with viable activated microglia/macrophages depicted in a scatter chart (A). A representative field of the counted sections is shown in B–D.
(TIF)

Figure S4 **MEGF10 expression is increased in cultures treated with dead cells compared to control cultures.** Cell cultures that have differentiated for 8 days, either received medium with or without (controls) apoptotic cells. Protein lysates were prepared after 1 or 3 days of incubation and used for Western blot analysis. The average increase, in three separate Western blots experiments, of MEGF10 expression (±SEM) compared to the controls without added cells is shown in the graph.
(EPS)

Video S1 **The astrocyte (yellow A) close to the injury immediately starts to struggle to bring the cell corpses (red stars) closer to the astrocytic cell body.** Scale bar = 20 µm.
(AVI)

Video S2 **After injury, the cells not cut through or in direct proximity of their nuclei, prune and withdraw from the injury site before regenerating and growing towards the laceration.** Injury increase neuronal motility, especially towards and along the cut, but without crossing the laceration. Scale bar = 20 µm, the dashed lines represent the scratch.
(AVI)

Video S3 **In the uninjured culture the neurons are less motile and move without an apparent goal.**
(AVI)

Video S4 **An engulfment cleft is formed in the highly vacuolized astrocyte (yellow A) in which the debris is ingested (red arrowhead).** The healthy neuron (green star) blebs and dies upon cell body contact with a dead cell close, but not engulfed, by the astrocyte (yellow A). Scale bar = 20 µm.
(AVI)

Video S5 **The healthy neuron (blue arrowhead) initially adheres to the dead cell via a process and then pulls it towards the cell body ultimately inducing apoptosis.** Following bystander cell death, both cell corpses are collected by a nearby astrocyte (that appear covered in dead cells in the film). Neurons (green stars), which are very motile after the injury, migrate several times over astrocytes with ingested dead cells without going into apoptosis. The phenomenon can also be seen in the last part of Video S2. Scale bar = 10 µm, the dashed lines represent the scratch.
(AVI)
