# Supplementary figures and images for: Engulfing Astrocytes Protect Neurons from Contact-Induced Apoptosis following Injury
Source: PLoS One. 2012 Mar 26;7(3):e33090. doi: 10.1371/journal.pone.0033090 (PMC3312890; doi:10.1371/journal.pone.0033090)

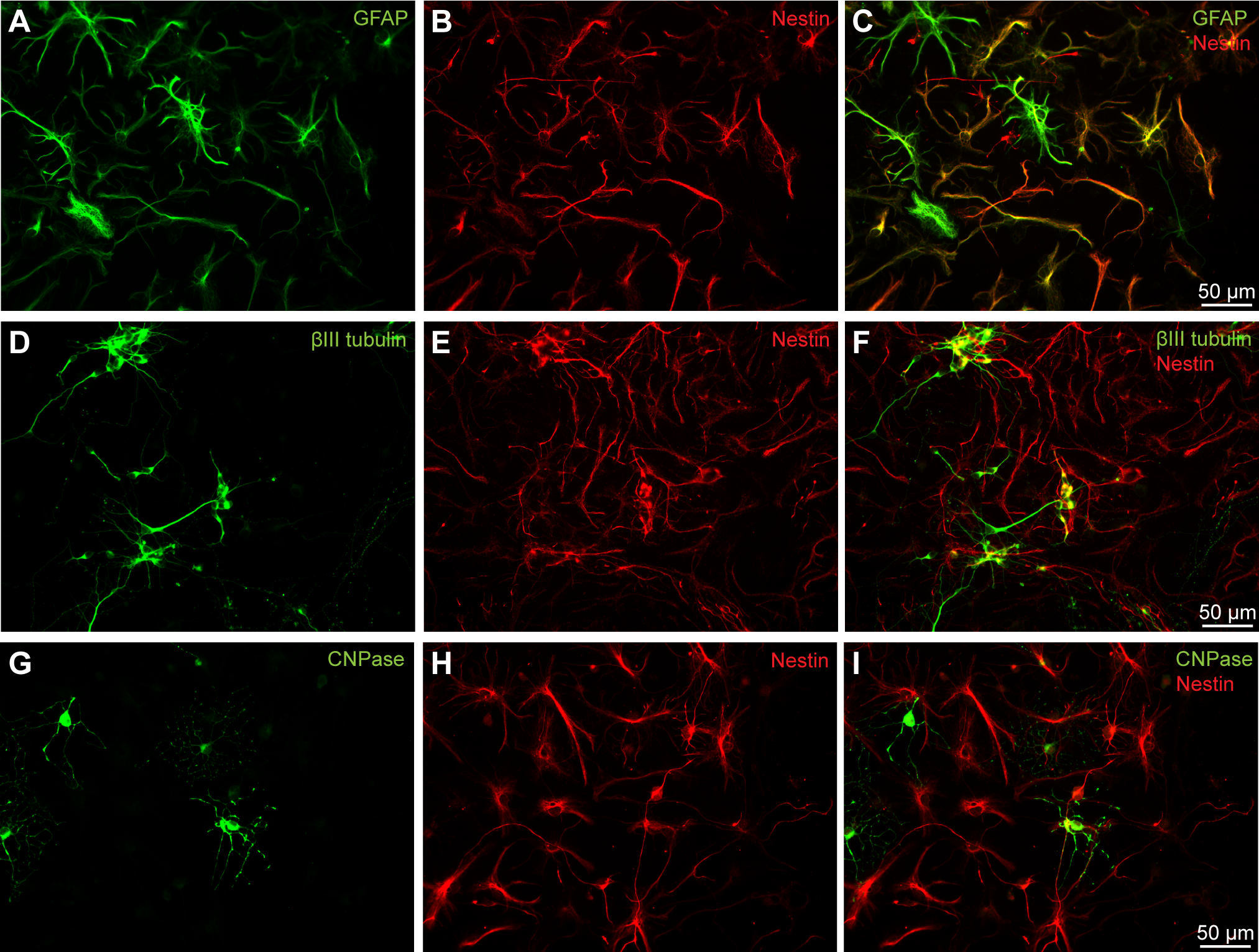

Supplement: Figure S1 — Astrocytes and immature neurons express nestin. Mixed cell cultures of neurons, astrocytes and oligodendrocytes were fixed in 4% PFA and stained with specific antibodies against nestin and (A–C) GFAP, (D–F) β III tubulin, and (G–I) CNPase. (TIF) [file pone.0033090.s001.tif]

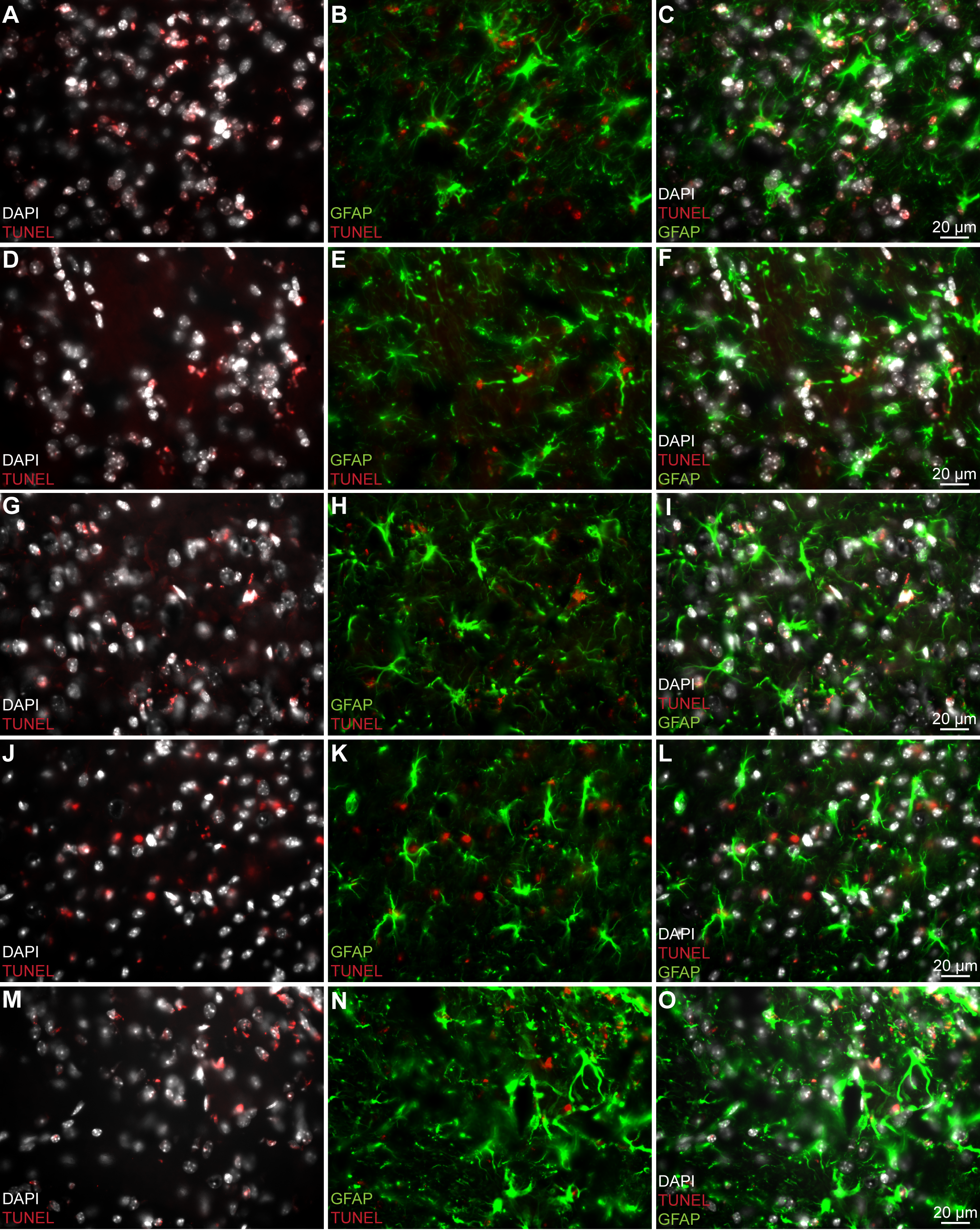

Supplement: Figure S2 — Examples of photos used for quantification of TUNEL and GFAP co-localization in vivo . Trauma was induced in five mice using a controlled cortical impact (CCI) injury model. Seven days post-injury, the animals were perfused and the brains were sectioned and stained with TUNEL label, DAPI and antibodies against GFAP. Five representative fields, A–C, D–F, G–I, J–L and M–O are shown. (TIF) [file pone.0033090.s002.tif]

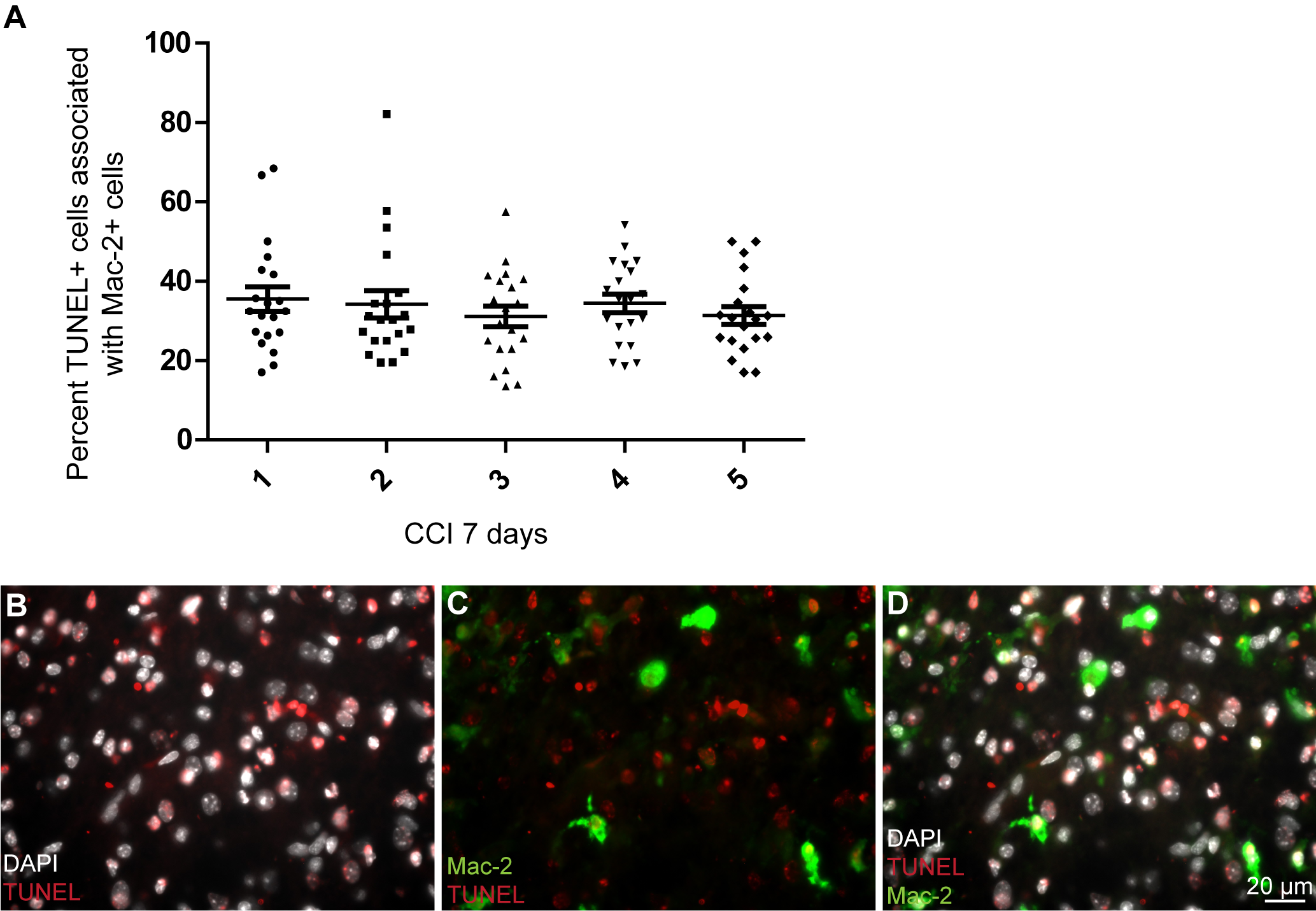

Supplement: Figure S3 — Quantification of TUNEL+ cells co-localized with Mac-2 positive cells in vivo . Trauma was induced in five mice using a controlled cortical impact (CCI) injury model. Seven days post-injury, the animals were perfused and the brains were sectioned and stained with TUNEL label, DAPI and antibodies against Mac-2. Ten representative fields per animal (n = 5) were counted and the percent TUNEL+ cells associated with viable activated microglia/macrophages depicted in a scatter chart (A). A representative field of the counted sections is shown in B–D. (TIF) [file pone.0033090.s003.tif]
